# Induced high-temperature ferromagnetism by structural phase transitions in strained antiferromagnetic *γ*-Fe_50_Mn_50_ epitaxial films

**DOI:** 10.1038/s41598-019-39949-x

**Published:** 2019-03-06

**Authors:** Younghun Hwang, Sungyoul Choi, Jeongyong Choi, Sunglae Cho

**Affiliations:** 1 Electricity & Electronics and Semiconductor Applications, Ulsan College, Ulsan, 44610 Republic of Korea; 2 Department of Physics and Energy Harvest-Storage Research Center (EHSRC), University of Ulsan, Ulsan, 44610 Republic of Korea

## Abstract

Strain effects in epitaxial films can substantially enhance individual functional properties or induce properties which do not exist in corresponding bulk materials. The bcc *α*-Fe_50_Mn_50_ films are a ferromagnetic with a Curie temperature between 650 K and 750 K, which do not exist in nature can be manipulated through the tensile strain. In this study, *γ*-Fe_50_Mn_50_ epitaxial films grown on GaAs(001) using molecular beam epitaxy are found to structural transition from the face-centered-cubic (fcc, *a* = 0.327 nm) *γ*-phase to the body-centered-cubic (bcc, *a* = 0.889 nm) *α*-phase. For *α*-Fe_50_Mn_50_ epitaxial films, ferromagnetism is accompanied by structural phase transition due to the tensile strain induced by the differences of the thermal expansion between the film and the substrate. Moreover, by realizing in epitaxial films with fcc structure a tensile strain state, phase transitions were introduced Fe-Mn alloy system with bcc structure. These findings are of fundamental importance to understanding the mechanism of phase transition and properties of epitaxial CuAu-I type antiferromagnetic alloy thin films under strain.

## Introduction

In the recent years, most of the paradigmatic concepts used in spintronics have been replicated substituting ferromagnets by antiferromagnets in critical parts of the devices^1–3^. The numerous research efforts directed to manipulate and probe the magnetic moments in antiferromagnetic materials have been gradually established a new and independent field known as antiferromagnetic spintronics^4–6^. Traditionally, antiferromagnetic-based magnetic storage devices such as spin-valve structures and magnetic tunnel junctions have been widely investigated in the emerging field of spintronics, in which the antiferromagnets provide pinning for a reference ferromagnetic layer due to an interfacial effect called an exchange bias^7,8^. However, recent studies have shown that antiferromagnetic materials play an important role in the manipulation of ferromagnets beyond the pinning effect due to the efficient spin transfer through the antiferromagnetic spin wave as well as the promising spin-orbit effect in the antiferromagnet^9,10^. Furthermore, the discovery of electrical switching of metallic antiferromagnets by spin-orbital torque has provided an example that the antiferromagnetic moment can be controlled more efficiently in a microelectronic device compared to a ferromagnetic material^11,12^. All these investigations have focused on metallic antiferromagnetic materials. For metallic antiferromagnetic materials, however, which play an irreplaceable role in traditional spintronic devices, direct electrical control remains challenging because of the screening effect by the surface charge. It is well known that the *γ*-Fe_50_Mn_50_ alloy thin films as an antiferromagnetic pinning layers are very useful in fabricating spin valve devices via exchange bias^13–16^. However, recently, it has been recognized that non-collinear antiferromagnetic spin textures result in Berry phases that profoundly change charge transport by generating anomalous Hall effects^17,18^. In addition, *γ*-Fe_50_Mn_50_ layers exhibit spin-Hall magnetoresistance and large inverse spin-Hall effect voltage, implying that the antiferromagnetic materials can be both spin current detector and generator^19–21^. These investigations open up new opportunities in developing the antiferromagnetic based spintronic devices. In this context, CuAu-I type metallic antiferromagnetic (AF) alloys such as *γ*-Fe_50_Mn_50_, Ir_50_Mn_50_, Pt_50_Mn_50_, and Pd_50_Mn_50_ are promising candidates for efficient and tunable electrical manipulation of ferromagnetic materials^22–25^. CuAu-I-type antiferromagnetic thin films are of significant interest due to their simple structure as well as the possibility of epitaxial growth on many ferromagnetic layers, which are crucial for many spintronics applications. Epitaxial growth in thin films, particular, provides an extremely powerful means of discovering for materials with promising properties by enabling the rapid preparation of new materials, and these properties can be tailored for a wide variety of emergent application^26–28^. Indeed, bcc phase Ni and Co do not exist as a bulk phase, for example, but were stabilized by epitaxial grown on the semiconductor substrate^29–31^. In addition, strain effects induced during epitaxial growth are of great current interest for improving materials properties. Strained epitaxial films are also able to enhance the saturation magnetization in ferromagnets^32,33^ and spontaneous polarization in ferroelectrics^34–36^ or can alter the Curie temperature(*T*_*C*_) of ferromagnetic and superconducting materials^37–39^. The properties of epitaxial films can distort the bulk structure and/or stabilize phases not present in the bulk material^40–42^.

In order to induce artificial strain on epitaxial thin films, we focus on the structural and the magnetic phase transitions of CuAu-I type *γ*-Fe_100−*x*_Mn_*x*_ alloys for efficient and tunable electrical manipulation of ferromagnetic materials. As is known, stoichiometric Fe_50_Mn_50_ alloy as a bulk and/or thin film has only an fcc *γ*-FeMn with an antiferromagnetic phase, while the bcc ferromagnetic phase does not exist. Therefore, one of the key issues is trying to prepare the bcc phase with Mn composition as same as possible to get more precise and reliable ferromagnetic phase. Electrical control of interfacial coupling has recently been successfully demonstrated in ferromagnetic/antiferromagnetic heterostructures, but manipulating ferromagnets from a single material may be used as an efficient spin current source.

In this work we demonstrate the methodology needed to create bcc ferromagnetic phase epitaxial thin films of Fe_100−*x*_Mn_*x*_ on crystalline substrates, which then allows the properties of epitaxial *γ*-Fe_100−*x*_Mn_*x*_ to be explored systematically. Our experimentally magnetic phase transition was shown to be accompanied by structural phase transition, it is dominated by the epitaxial strain effect from the substrates.

## Results and Discussion

**Figure 1:**
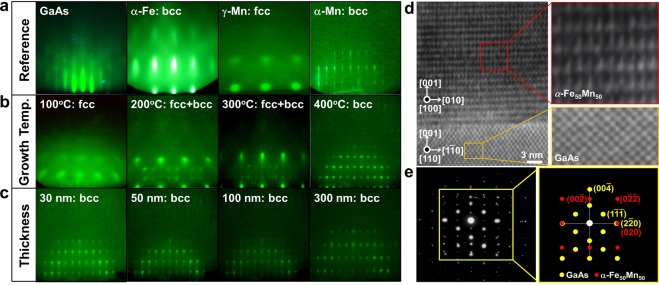
(**a**) RHEED patterns of 20 nm pure bcc-Fe, fcc *γ*-Mn, and bcc *α*-Mn films epitaxially grown on GaAs(001) substrates, taken with the incident electron beam along the substrate [$1\bar{1}0$] direction. (**b**) RHEED pattern of 30 nm Fe_50_Mn_50_ films grown on GaAs(001) with various growth temperature of 100, 200, 300 and 500 °C, respectively. (**c**) RHEED patterns for epitaxial *α*-Fe_50_Mn_50_ films grown at 400 °C with various thicknesses of 30 nm, 50 nm, 100 nm, and 300 nm, respectively. (**d**) High-resolution TEM images of the 100 nm *α*-Fe_50_Mn_50_ film (up) grown on GaAs (down) at 400 °C. (**e**) (left) SAED pattern of a 100 nm *α*-Fe_50_Mn_50_ film and (right) simulated diffraction pattern of *α*-Fe_50_Mn_50_(001)[110]||GaAs(001)[100] along the [110] direction of GaAs substrate.

**Figure 2:**
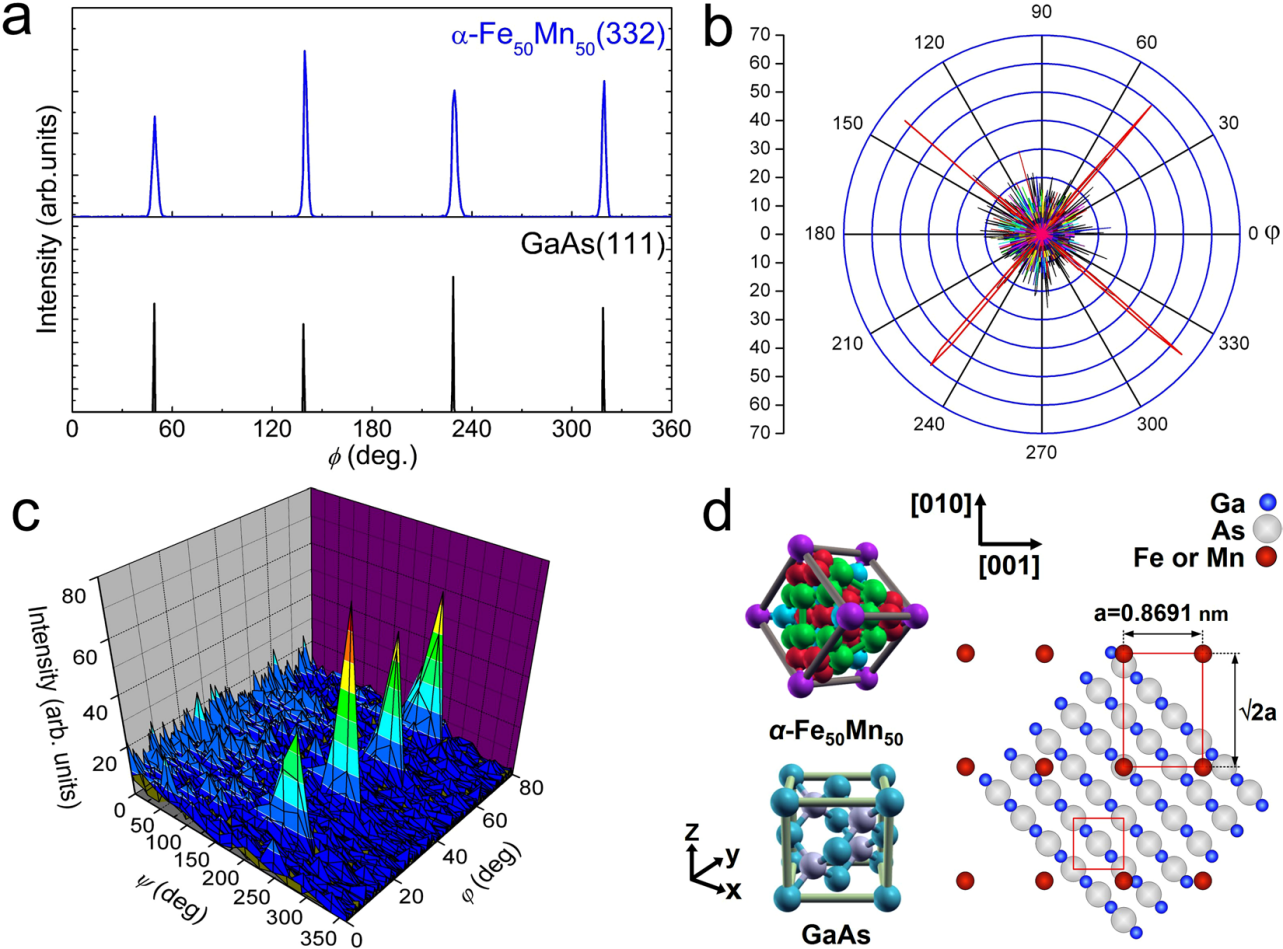
(**a**) XRD *ϕ*-scans of Fe_50_Mn_50_(332) and GaAs(111) planes of *α*-Fe_50_Mn_50_ film grown on GaAs(100). (**b**,**c**) pole figures of epitaxial *α*-Fe_50_Mn_50_ thin film. The bcc *α*-Fe_50_Mn_50_ film grown on GaAs(100) showing an in-plane 45° rotation between the epilayer and substrate. The *ϕ*-scans from the *α*-Fe_50_Mn_50_(332) and GaAs(111) plane were obtained under the fixed of *ψ* = 25.24°, 2*θ* = 47.83°, and *ψ* = 54.74°, 2*θ* = 27.30°, respectively. (**d**) 3D atomic configuration of *α*-Fe_50_Mn_50_ and GaAs. Epitaxial relationships between *α*-Fe_50_Mn_50_ and GaAs: (001)[110]_*α*-FeMn_ ||(001)[100]_GaAs_.

Figure 1a shows in site RHEED patterns for a clean GaAs(100) surface, with an electron beam along the azimuth of [$1\bar{1}0$]. For comparison, the diffraction patterns of pure fcc *γ*-Mn, bcc-Fe, and bcc *α*-Mn films epitaxially grown on GaAs(001) are also presented in Fig. 1a. The difference between these three patterns is quite obvious: The fcc and bcc phases have a square- and rectangle-like patterns^43,44^, while the *α*-Mn film has a streaky-like pattern^45^. Figure 1b shows a RHEED pattern of 30-nm Fe_50_Mn_50_ films grown on GaAs(001) at different growth temperatures. The film grown at 100 °C has square-like pattern, which is consistent with *γ*-Mn pattern. However, as the growth temperature surpasses 100 °C, some other extra spots appear as superposed on *γ*-Mn diffraction pattern, as shown in Fig. 1b. It might seem at first glance that two-crystalline growth is starting to develop at this stage, but this is actually a transition stage where *α*-FeMn phase grows on the top of *γ*-FeMn phase. When the growth temperature between the 200 and 300 °C, assuming that the coexistence of fcc *γ*- and bcc *α*-phase where Fe_50_Mn_50_ structure. Following the evolution of the RHEED pattern as a function of temperature, we first found that a new pattern starts to appear at 400 °C as the substrate spots fade away. This new diffraction pattern becomes completely dominant at above 30 nm. Figure 1c shows the RHEED patterns for epitaxial *α*-Fe_50_Mn_50_ films grown at 400 °C with various thicknesses of 30 nm, 50 nm, 100 nm, and 300 nm, respectively. Qualitatively speaking, it is clear from the figure that the RHEED patterns change gradually from streaky-like to rectangle shapes as the film develops. It is obvious from such a well-ordered pattern that the FeMn overlayer grows in a single-crystalline structure on the GaAs substrate at this stage. Surprisingly, the structure of *α*-Fe_50_Mn_50_ films grown at 400 °C is purely different from its previously grown *γ*-Fe_50_Mn_50_ thin films^46,47^. These results are understandable because the higher growth temperature is sufficient to give enough mobility to Fe and Mn atoms to overcome the lattice mismatch for epitaxial growth. This claim was supported by high resolution transmission electron microscope (HRTEM) images and it is selected area electron diffraction (SAED) pattern. Here, we focused on the emergence and growth of *α*-Fe_50_Mn_50_ with bcc phase. Note that the absence of any superstructure reflection in the SAED pattern and the absence of defects in the HRTEM images indicate that no clusters were present in 100 nm-thick *α*-Fe_50_Mn_50_ thin film grown at 400 °C. Figure 1d shows a cross-sectional HRTEM image of Fe_50_Mn_50_ layer on the GaAs(001) substrate with the electron beam along the [110] direction. The zone axes of the Fe_50_Mn_50_ layer and the GaAs substrate were <$1\bar{1}0$> and <110>, respectively. The unit cells in both regions are drawn, from which the Fe_50_Mn_50_ film is unambiguously identified to have bcc *α*-Mn structure with a lattice constant of 0.869 nm. The relative orientation between the film and substrate crystals is predicted the 45°-rotated configuration of *α*-Fe_50_Mn_50_(001)[110]||GaAs(001)[100] with a unit cell matching ratio of 2^1/2^*a*_FeMn_:2*a*_GaAs_ (8.68%). Although some interface dislocations appeared (typically, one dislocation was observed every 20 atomic planes) to reduce the strain energy induced by the large lattice mismatch between film and substrate, our result reveals that high quality epitaxial growth of *α*-Fe_50_Mn_50_ film grown on GaAs at 400 °C could be realized. Figure 1e shows the SAED pattern covering the whole region, whit the zone axis along the [110] direction of GaAs. Comparing the simulated diffraction pattern of *α*-Fe_50_Mn_50_(001)[110]||GaAs(001)[100] in Fig. 1f, we can confirm the single-crystalline nature of the sample and the in-plane crystallographic relationship expected by the *in situ* RHEED observation. Consequently, these measurements clearly demonstrate that the epitaxial growth of *α*-Fe_50_Mn_50_ on GaAs is realized. In order to characterize more precisely the epitaxial layer grown on GaAs substrate, *ϕ*-scan and pole figure measurements were performed. Figure 2 shows the *ϕ*-scans and pole figures, respectively. The four symmetric peaks in the *ϕ*-scan of the film indicates the specific in-plane epitaxial growth pattern of *α*-Fe_50_Mn_50_(110) film on the GaAs(001). A pole figure scans of *α*-Fe_50_Mn_50_(332) and GaAs(111) reflection showed four diffraction peaks, owing to the diffraction geometry of the (110)-oriented *α*-Fe_50_Mn_50_ films. The film and the substrate unit cells are rotated by 45° in-plane. From above mentioned the *ϕ*-scan and pole figure studies, GaAs(001) with zinc-blend structure together with the corresponding Miller indices were illustrated in Fig. 2d. The geometry of the epitaxial *α*-Fe_50_Mn_50_ growth are expected, i.e. bcc *α*-phase with (001)[110]_film_||(001)[100]_GaAs_.

**Figure 3:**
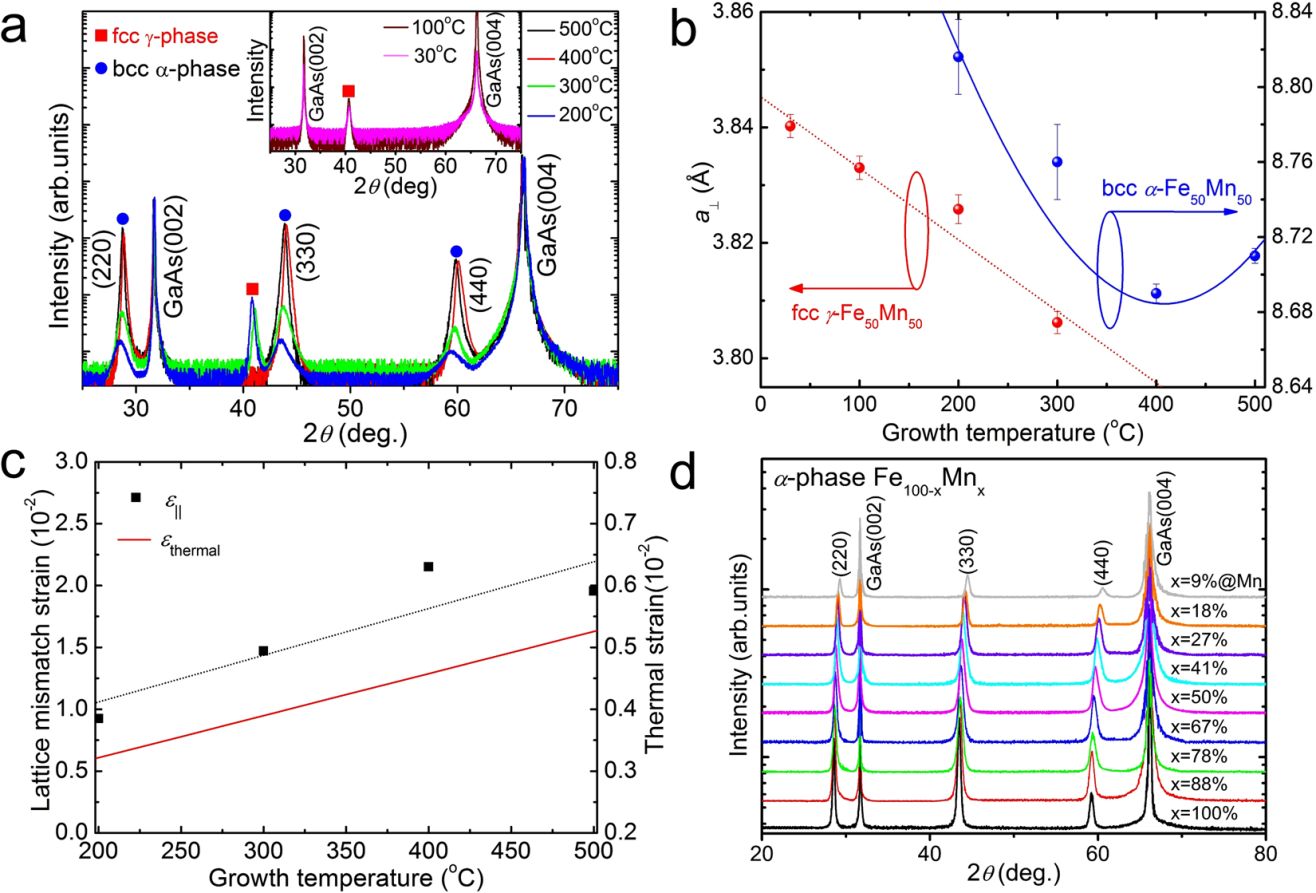
(**a**) *θ*–*2θ* XRD patterns for 100 nm Fe_50_Mn_50_ films grown at different growth temperature. The solid square and circle symbols correspond to *γ*- and *α*-phase diffraction peaks, respectively. (**b**) The perpendicular lattice constants (*a*_⊥_) calculated from the XRD data for both the *γ*-(red circles) and *α*-Fe_50_Mn_50_ (blue circles) phases, respectively. (**c**) Strain due to thermal expansion coefficient and lattice mismatch at different growth temperature. (**d**) *θ*-2*θ* XRD patterns of *α*-phase Fe_100−*x*_Mn_*x*_ alloy films for the whole range of Mn concentration 0.09 ≤ *x* ≤ 1 grown at 400 °C.

Figure 3a shows the *θ*-2*θ* XRD peak positions for both fcc *γ-* and bcc *α*-phases in the Fe_50_Mn_50_ films as a function of growth temperature. For *T* ≤ 100 °C, the diffraction peak can be indexed to the *γ*-phase, consistent with an earlier structural study of *γ*-Fe_50_Mn_50_^48,49^. However, at intermediate growth temperatures 200 ≤ *T* ≤ 300 °C, not only the *γ*-phase but also three new diffraction peaks were observed, which characteristic peak correspond to the bcc *α*-phase Mn structure (JCPDS 65–3164)^50^. When the growth temperature is higher than 400 °C, the *γ*-Fe_50_Mn_50_ diffraction pattern disappears and only the (220) and (330), and (440) characteristic peaks corresponding to *α*-phase Fe_50_Mn_50_ are observed. Until now, this result has not reported experimental and theoretical studies with the single *α*-phase Fe_50_Mn_50_ thin films. This suggests that the crystalline structure of the grown film evolved gradually from the *γ*- to the *α*-Fe_50_Mn_50_ phase with increasing growth temperature and that the temperature of sample fabrication plays an important role in the phase transition of epitaxial Fe_50_Mn_50_ films. Figure 3b shows the lattice constants perpendicular to the film (*a*_⊥_) plane calculated from the XRD data for both the *γ*- and *α*-Fe_50_Mn_50_ phases as a function of growth temperatures. For *γ*-phase, the lattice constants were larger than the known bulk *γ*-Fe_50_Mn_50_ value of 0.3629 nm and decreased with increasing growth temperature, up to 300 °C. This indicates that compressive strain acts in a direction parallel to the film plane and gradually becomes weaker as the with growth temperature increases. For *α*-phase, on the contrary, lattice constants were smaller that the known bulk value of 0.8911 nm and decreased with increasing growth temperature up to 500 °C, indicating tensile strain in films which becomes stronger with increased growth temperature. Assuming that the Fe_50_Mn_50_ layer is strained elastically, strain perpendicular to the plane as a function of growth temperature was calculating using *ε*_*zz*_ = (*a*_⊥_ − *a*_0_)/*a*_0_, where *a*_⊥_ and *a*_0_ stand for thin film and bulk lattice constants. In addition, the biaxial strain (in-plane strain; *ɛ*_||_) is related to the strain perpendicular to the film plane is expressed by *ɛ*_||_ = − (C_11_/2C_12_)·*ε*_*zz*_, where C_11_ and C_12_ are elastic stiffness coefficient of Fe_50_Mn_50_, and the values used in the calculation are C_11_ = 170 GPa and C_12_ = 98 GPa^51^. A positive (or negative) strain value indicates an in-plane tensile (or compressive) strain for the film. The growth temperature dependence of in-plane strain (*ɛ*_||_) for the Fe_50_Mn_50_ film estimated by x-ray analysis, and it is confirmed that the strain increases as the growth temperature increases (Fig. 3c). On the other hand, considering only the strain due to lattice mismatch, the observed results at different growth temperature are opposite than what we have expected because the lattice constant of Fe_50_Mn_50_ thin film is smaller(*γ*-phase) or larger(*α*-phase) than that of GaAs (*a* = 0.5654 nm). In particular, we have focused on the *α*-Fe_50_Mn_50_ thin films, which undergo phase transition from *γ*- to *α*-phase. Strain due to lattice mismatch is considered to be almost relaxed in the case of thin films that are much thicker than the critical thickness. In other words, the sample thickness used in this study is 100 nm, which is considerably thicker than the critical thickness of 5 nm calculated by a simple beam equation, so that the lattice mismatch strain at the interface is considered to be almost relaxed. Therefore, the observed tensile strain may be due to the difference in thermal-expansion coefficients between Fe_50_Mn_50_ (*α*_Fe50Mn50_ = 11.5×10^−6^ K^−1^) and GaAs (*α*_GaAs_ = 5.4×10^−6^ K^−1^Ga) at 300 K. In the heterojunction structure, the thermal expansion coefficients of the two materials are different, and the thermal strain due to the difference in thermal expansion coefficient is expressed by *ε*_thermal_ = *K*(*α*_FeMn_ − *α*_GaAs_)Δ*T*, where *K* describes the value of the lattice relaxation and Δ*T* is the temperature difference between the growth and the measured temperatures. For films that are thick enough, *K* might be set to 1 because the strain due to the lattice mismatch could be assumed to be fully relaxed. The thermal strain at room temperature for the sample grown at 400 °C is estimated to be approximately 2.3×10^−3^, using 300 K constants. As growth temperature increase, the thermal strain increases, resulting in a decrease in lattice constants with growth temperature. The Fe_50_Mn_50_ films grown on GaAs are tensely strained due to the thermal expansion coefficients (Supplementary Fig. S1). During growth, the lattice mismatch strain is relaxed at above 5 nm, and as the sample is cooled to room temperature after growth, both the film and the substrate are shrunk. In our system, thermal strain due to the difference in thermal expansion coefficients between the film and the substrate is not negligible. Therefore, observed tensile strain may be due to the lattice distortion by difference in thermal expansion coefficients between the *α*-Fe_50_Mn_50_ film and the GaAs. On the other hand, according to the phase diagram of Fe-Mn alloys, the *α*-phase is stable only when the Mn composition is less than 5%. Therefore, it is necessary to prepare a bcc-phase with Mn concentration as high as possible, in order to investigate the influence of Mn ions in a wide range of compositions as well as get more reliable magnetic measurements. The Fe_100−*x*_Mn_*x*_ alloys in the *α* phase (*α*-FeMn) have a bcc lattice structure which is observed up to about 20% Mn. In this regard, we investigated the *θ*-2*θ* XRD patterns of Fe_100−*x*_Mn_*x*_ alloy films for the whole range of Mn concentration grown at 400 °C (Fig. 3d). The bcc *α*-phase Fe_100−*x*_Mn_*x*_ phase was obtained in the whole Mn concentration ranges, and these new structural phase transitions are not reported up to now, consistent with the results of magnetic phase transitions of Fe_100−*x*_Mn_*x*_ films. Note that bulk Fe_100−*x*_Mn_*x*_ alloys exhibit the *γ*-Fe_100−*x*_Mn_*x*_ phase with an fcc structure for the Mn concentration 30% < *x* < 60%. Whereas, Fe_100−*x*_Mn_*x*_ alloy films grown at low temperatures (less than 100 °C) exhibited fcc *γ*-FeMn phases at the Mn concentration *x* > 40% (Supplementary Fig. S2), which consistent with previous results.

**Figure 4:**
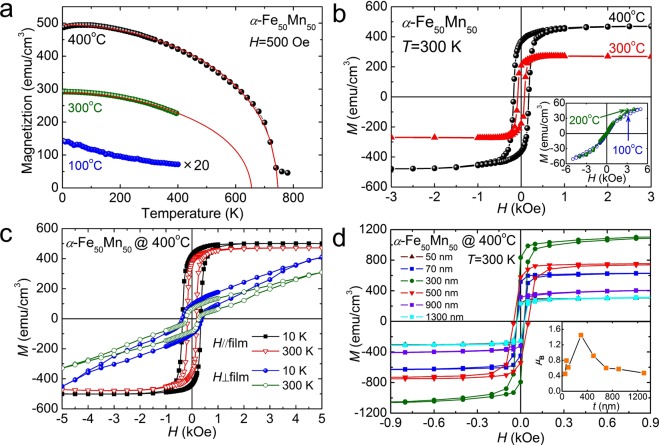
(**a**) Temperature-dependent magnetization (*M*) of epitaxial Fe_50_Mn_50_ thin films grown at 100, 300 and 400 °C in a magnetic field of 500 Oe. The fit *M*(*T*) = *M*_S_[1 − (*T*/*T*_C_)^2^]^1/2^ gives *T*_C_ = 650 and 750 K at 300 and 400 °C, respectively. (**b**) Magnetic hysteresis loops with magnetic field parallel to the film plane at 300 K. The inset shows the *M*–*H* curves of Fe_50_Mn_50_ films grown at 100 and 200 °C, respectively. (**c**) The magnetic field parallel and perpendicular to the film plane curves of *α*-Fe_50_Mn_50_ films at 10 and 30 K. Saturation is more easily attained with a parallel magnetic field. (**d**) Thickness-dependent hysteresis loops at 300 K for *α*-Fe_50_Mn_50_ thin films grown at 400 °C. The highest magnetic moment corresponds to a 300 nm thick-film of *α*-Fe_50_Mn_50_ as shown in the inset.

Figure 4a shows the temperature (*T*)-dependent magnetization (*M*) of 100 nm-thick Fe_50_Mn_50_ films as a function of growth temperature between 5 and 800 K in a magnetic field *H* = 500 Oe. The diamagnetic contribution of the sample holder was independently measured by removing the sample, and this background has been subtracted. For *γ*-Fe_50_Mn_50_ grown at 100 °C, magnetization is similar to paramagnetic or AF behaviours, but the weak ferrimagnetic hysteresis loop was observed at 300 K, unlike previously obtained bulk results for the *γ*-Fe_50_Mn_50_^52^. The other side, in case of *α*-Fe_50_Mn_50_ grown at 300 and 400 °C, the magnetization as a function of temperature can be fitted well to *M*(*T*) = *M*_S_[1-(*T*/*T*_C_)^2^]^1/2^ ^53^. The Curie temperature (*T*_C_) obtained from the fit to equation is 650 and 750 K at 300 and 400 °C, respectively. A phase transition from magnetically disordered (paramagnetic) states to a magnetically ordered (ferromagnetic) state occurs due to the structure transformations induced by strain. The saturation magnetization (*M*_S_) is 268 and 470 emu/cm^3^ at 300 K for *α*-Fe_50_Mn_50_ films with growth temperature of 300 and 400 °C, respectively. Figure 4b shows the magnetization loops of 100 nm *α*-Fe_50_Mn_50_ film as a function of the magnetic field applied parallel to the film plane at 300 K as a function of growth temperature. The magnetization loops show the hysteretic behaviour in the films grown at 300 and 400 °C. The saturated magnetization (*M*_s_) was 289 and 480 emu/cm^3^ at 300 K in the samples grown at 300 and 400 °C. These values are in good agreement with those obtained by fitting the *M*–*T* data. In Fig. 3b shows that the coercive field is 75 Oe at 300 K with sample grown at 300 °C and the coercive field increases with increasing growth temperature. Those indicate that the spin-spin interaction should become enhanced with increasing growth temperature, which is consistent with the above behaviour of the saturation magnetization. We also find that, weak ferrimagnetic ordering with coercive fields of 186 and 225 Oe at 10 K in the films grown at 100 and 200 °C (inset in Fig. 4b), in contrast with the AF ordering seen in bulk^51^. From the magnetization curves, the rectangular hysteric behaviour was observed in a magnetic field parallel to the film as mentioned (see Fig. 4c), while the nonrectangular behaviour was observed in the perpendicular to the film plane, which indicates that the presence of the magnetic anisotropy. Figure 4d shows the magnetic hysteretic loops of *α*-Fe_50_Mn_50_ film grown at 400 °C with various thicknesses under parallel magnetic fields. The magnetic moments observed at 300 K ranged between 129 and 1121 emu/cm^3^; the highest magnetic moment for *α*-Fe_50_Mn_50_ was observed in a 300 nm thick film. Thickness dependence of saturated magnetizations for *α*-Fe_50_Mn_50_ samples at 300 K, we calculated the average magnetic moments per magnetic ion in the system (i.e., for both Fe and Mn) for thickness = 50 nm, 100 nm, 300 nm, and 1300 nm to be, respectively, 0.45 *μ*_B_, 0.62 *μ*_B_, 1.45 *μ*_B_, and 0.47 *μ*_B_, as shown in the inset in Fig. 3d. Here we noticed that the spin arrangement between Mn and Fe could not be distinguished from the saturated magnetization of *α*-Fe_50_Mn_50_ films. Bulk fcc Fe_50_Mn_50_ disordered alloy is known to has a non-collinear antiferromagnetic spin structure, which is characterized by four different sublattices in which the spins point along four different <111> direction. However, if the distances between the Fe and/or Mn ions are changed as a result of the structural phase transition due to the epitaxial strain effect, which might result in ferromagnetic ordering above room temperature. These results suggest that the epitaxial strain imposed by the substrates can give rise to behavior distinct from those obtainable through bulk states, essentially, it creates new materials from old elements and greatly increases the properties of various magnetic materials. Similarly, bulk crystals are not superconducting or ferromagnetic at ambient pressure or under high pressure, but superconductivity or ferromagnetism are reported due to epitaxial strain in thin films^41,42,45^. Our experiment data of high temperature ferromagnetism induced via epitaxial strain play a key role for a better understanding of the structural phase transition of CuAu-I type antiferromagnetic materials and the ferromagnetic mechanism of this material categories.

**Figure 5:**
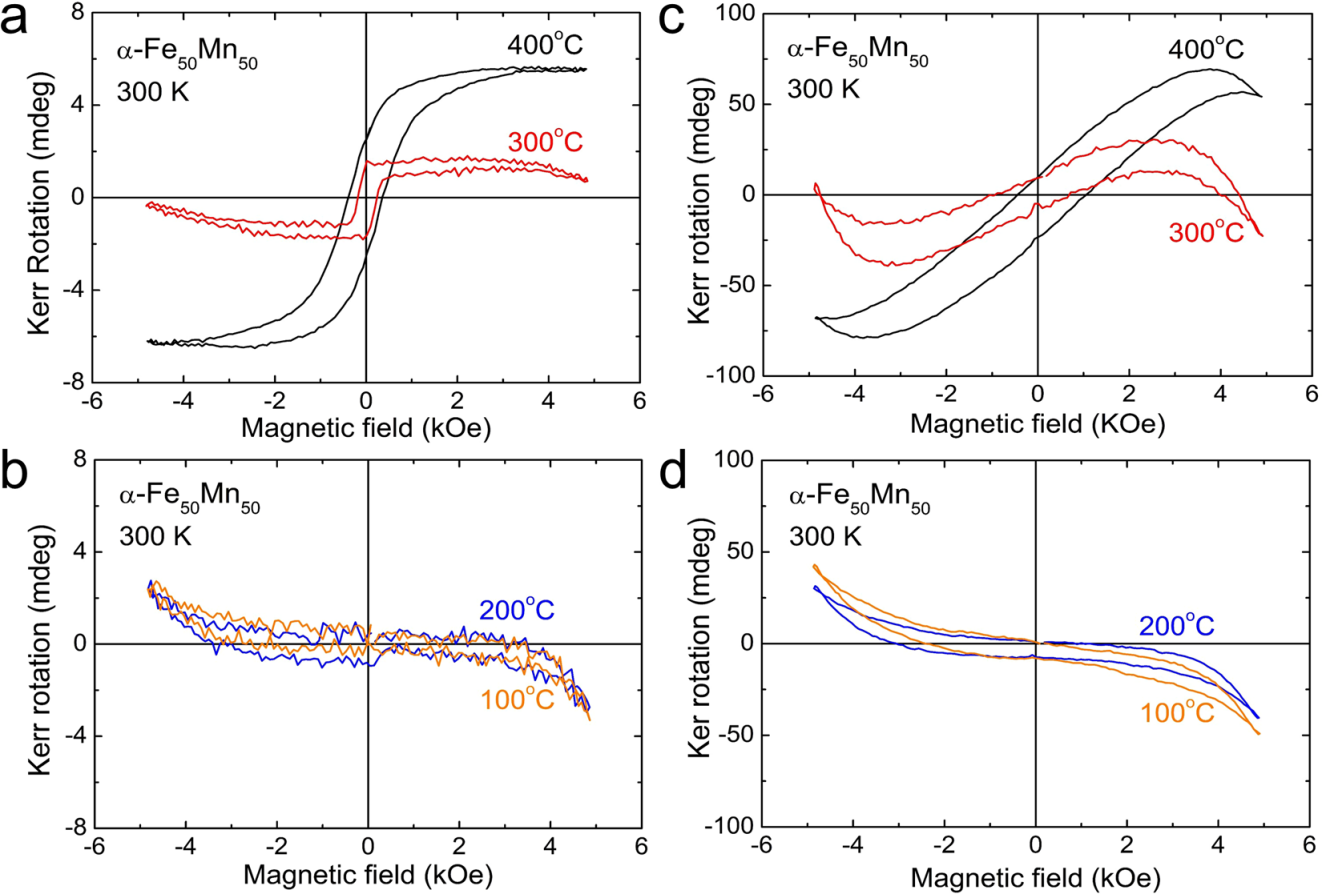
(**a**,**b**) Hysteresis loops of Kerr rotation for the longitudinal configuration of the Fe_50_Mn_50_ films grown at 100, 200, 300, and 400 °C. (**c**,**d**) Hysteresis loops of Kerr rotation for the polar configuration of the Fe_50_Mn_50_ films grown at 100, 200, 300, and 400 °C.

We performed the magneto-optical Kerr effect (MOKE) measurements on both linearly- and elliptically-polarized incident light and recorded the Kerr rotation angle and ellipticity of the material as a function of the applied magnetic field. The general behavior of the results is consistent with the magnetization measurements performed using the SQUID. The Kerr rotation angle and the ellipticity were obtained in the longitudinal and the polar configurations at 300 K. For the samples grown at 300 and 400 °C, the magnetic field induced longitudinal Kerr rotation angle of the polarized light reflected from the surface of *α*-Fe_50_Mn_50_ epitaxial films displayed rectangular-like shaped loops with a coercivity 230 Oe and 380 Oe, respectively (see Fig. 5(a)). On the other hand, polar Kerr rotation showed non-rectangular hysteric behaviors. The normal component of the magnetization is seen in the polar geometry of MOKE, whereas the in-plane component is sensed in the longitudinal geometry of MOKE. When the normal component of the remanent magnetization vanishes, the hysteresis loop in the polar MOKE reduces to an S-like shaped loops (see Fig. 5(c)). α-Fe_50_Mn_50_ epitaxial films have magnetic anisotropic, the magneto-optical response is asymmetric, apparently violating the invariance of the hysteresis loop. The magnetic properties of *γ*-Fe_50_Mn_50_ are similar to those of paramagnetic or antiferromagnetic, but for *γ*-Fe_50_Mn_50_ grown at 100 and 200 °C (Fig. 5(b,d)), a weak magnetic hysteresis loop at 300 K was observed, unlike the results of the well-known *γ*-FeMn. The observed Kerr rotation hysteresis loops clearly suggest that the epitaxial *α*-Fe_50_Mn_50_ films are ferromagnetic up to and above room temperature. The above MOKE results agree well with the ferromagnetic order at high temperature obtained in the SQUID measurements.

From the above mentioned the magnetic properties, after having investigated the conditions for the appearance of ferromagnetism, we now try to understand its origin: is it intrinsic or extrinsic? The quality of the epitaxy is confirmed by high resolution TEM as shown in Fig. 1d, and no clusters or secondary phases can be observed within the detection limits of the instrument. This is consistent with the high resolution x-ray diffraction patterns, which shows only diffraction periodicities relating to the *α*-Fe_50_Mn_50_ crystalline phase. In order to confirm the presence or absence of clusters and secondary phases, for the *α*-Fe_50_Mn_50_ film grown at 400 °C, we conducted the XPS analysis as a function of the etching depth (Supplementary Fig. S3). The depth XPS results show that the added Fe into Mn does not form clusters and/or secondary phases, consistent with the results of HRXRD and HRTEM analyses.

**Figure 6:**
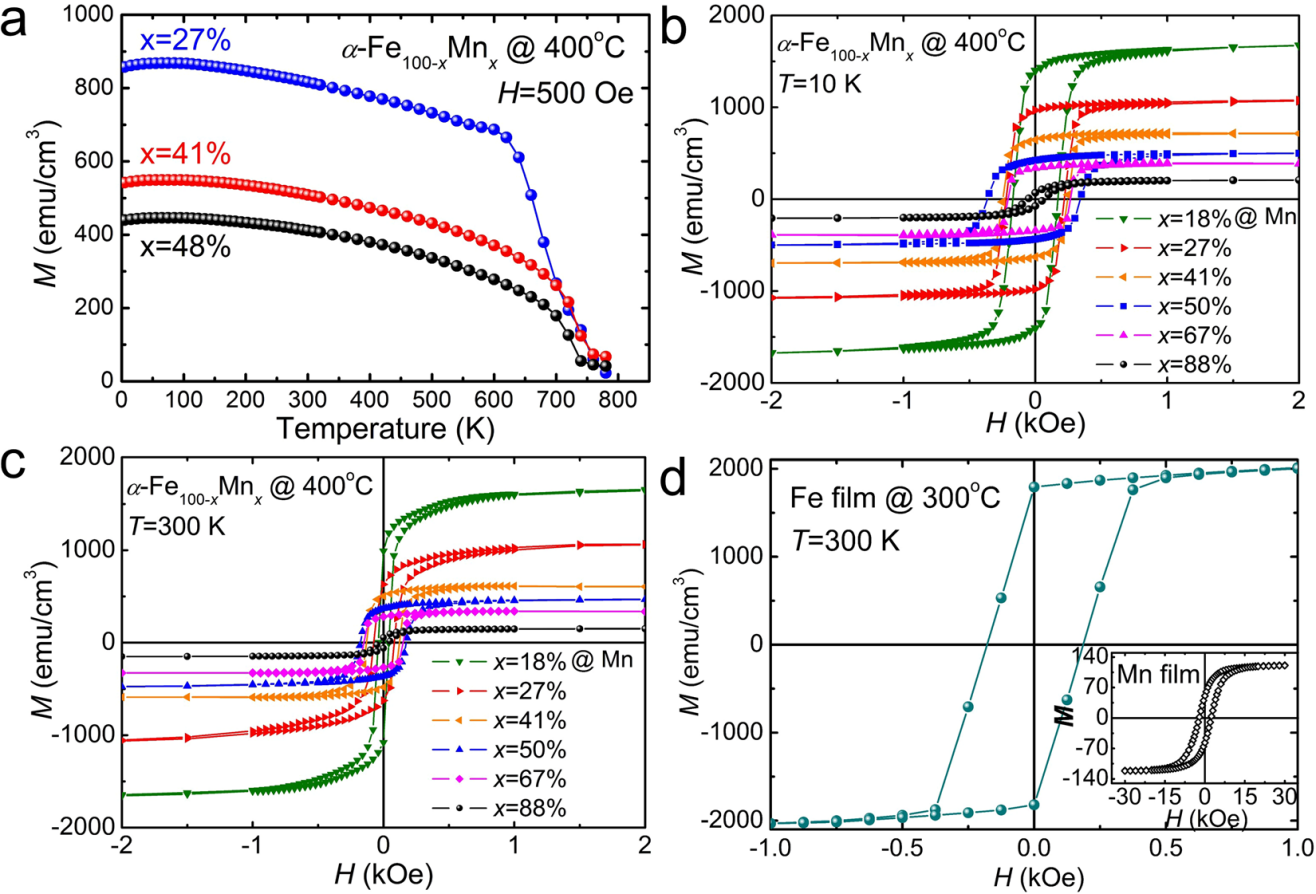
(**a**) Temperature-dependent magnetization (*M*) of bcc *α*-Fe_100−*x*_Mn_*x*_ films grown at 400 °C, with Mn concentrations *x* = 27% and 41% measured in a field of 500 Oe parallel to the film plane. The shapes of the magnetization vs temperature curves show ferromagnetic Brillouin function behavior. (**b**,**c**) Magnetic field dependence of magnetization of bcc *α*-Fe_100−*x*_Mn_*x*_ samples with Mn concentrations *x* = 18%, 27%, 41%, 50%, 67%, and 88% at 10 K and 300 K. (**d**) Magnetic hysteresis loops of pure bcc *α*-Fe film at 300 K. The inset shows the *M* vs *H* for *α*-Mn film at 300 K, which exhibits ferromagnetism even at the value without Fe ion.

**Figure 7:**
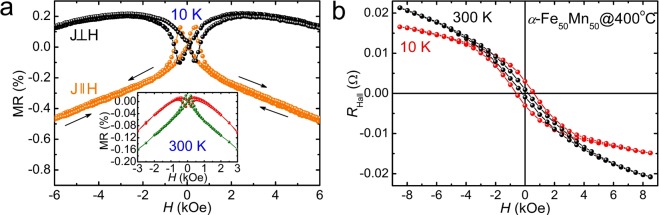
(**a**) Magnetoresistance of the *α*-Fe_50_Mn_50_ film at 10 K (300 K in inset). The MR is shown in two orthogonal directions, *J*||*H* and *J*⊥*H*, where *J* and *H* are the current density and the applied field, respectively. (**b**) The anomalous Hall resistances, *R*_Hall_, at 10 and 300 K, of the *α*-Fe_50_Mn_50_ film grown at 400 °C.

The ferromagnetic ordering of *α*-Fe_100−*x*_Mn_*x*_ alloys is bcc lattice structure, it has only been observed up to about 20% Mn. However, as a result of the structural phase transitions due to the epitaxial strain effects, Fe_50_Mn_50_ epitaxial films proved to exhibit ferromagnetism higher than room temperature. Based on this experimental basis, we have investigated the magnetic behavior of Fe_100−*x*_Mn_*x*_ films for the whole range of Mn concentration 0 ≤ *x* ≤ 100% grown at 400 °C (Fig. 6). Figure 6a shows the temperature dependent magnetization of *α*-Fe_100−*x*_Mn_*x*_ samples, with Mn concentrations *x* = 27% and 41% measured in a field of 500 Oe, indicating that the Fe_100−*x*_Mn_*x*_ films with Mn concentration *x* > 20% are ferromagnetic ordering above 750 K, consistent with our conclusion that the presence of epitaxial strain is responsible for the observed ferromagnetic behavior. For completeness, in Fig. 6b,c we show magnetization as a function of magnetic field to the film plane for *α*-Fe_100−*x*_Mn_*x*_ alloy films at 10 K and 300 K obtained from SQUID measurements. The observed hysteresis loops and the magnetization profiles clearly suggest that all samples are ferromagnetic up to and above room temperature. We have also found that the saturation magnetization increases as the Fe content increases, while the Mn content decreases, indicating that Fe and/or Mn ions play a key role in establishing ferromagnetic order observed in epitaxial strained Fe_100−*x*_Mn_*x*_ alloy films. These new ferromagnetic phases are significantly different from the bulk cases reported previously (Supplementary Fig. S4). The discovery of new magnetic and structural phase transitions within the same composition of epitaxial Fe_100−*x*_Mn_*x*_ thin films induced by strain, greatly increase the variety of materials as well as provide an opportunity to explore a new phenomenon in CuAu-I type antiferromagnetic spintronic materials. Using the values of saturation magnetizations measured on the *α*-Fe_100−*x*_Mn_*x*_ samples at 300 K, we calculated the average magnetic moments per magnetic ion in the system (i.e., for both Fe and Mn). The intriguing fact is, the average magnetic moment determined from the saturated magnetizations of epitaxial Mn and Fe thin films grown at 400 °C was 0.2 *μ*_B_ in pure Mn, increased with Fe content, and was 2.12 *μ*_B_ in pure Fe at 300 K (Fig. 6d). Figure 7a shows the magnetoresistance (MR) versus magnetic field curves for *α*-Fe_50_Mn_50_ grown at 400 °C between 10 and 300 K, respectively. The MR is shown in two orthogonal directions, *J*||*H* and *J*⊥*H*, where *J* is the current density and *H* is the applied field. The sample showed a negative MR at temperatures below *T*_C_ for all orientations of the current and field, as has previously been attributed to the suppression of spin fluctuations^54^. Strong hysteresis, understood to be a property of ferromagnetic materials, was observed at low field strengths in the MR curves. The hysteresis effect itself is related to magnetization hysteresis, indicating the presence of spin-polarized electron carriers in *α*-Fe_50_Mn_50_. The peak in the MR hysteresis corresponds to the coercive field. As the applied field was increased beyond saturation, the magnetization of the sample did not change significantly, but magnetization rotation occurred, contributing to a larger negative MR when *J* was parallel to *H*. This increase in negativity arises from the anisotropy magnetoresistance (AMR) effect^55^, the change in angle between the magnetization and the current due to the magnetization rotation observed at high fields being weaker than the anisotropy field. Magnetic field dependence of the Hall resistance at 10 and 300 K for *α*-Fe_50_Mn_50_ film grown at 400 °C (Fig. 7b). The hysteresis and remanence of Hall resistances were observed below *T*_C_, which is consistent with the results of observations via SQUID, MOKE, and MR measurements.

## Conclusions

In summary, we have achieved for the firstly single crystalline a ferromagnetic bcc *α*- Fe_50_Mn_50_ films on GaAs substrate. This suggests that the crystalline structure of the grown film evolved gradually from the *γ*- to the *α*-Fe_50_Mn_50_ phase with increasing growth temperature and that the temperature of sample fabrication plays an important role in the phase transition of epitaxial Fe_50_Mn_50_ films. The bcc *α*-Fe_50_Mn_50_ is also ferromagnetic with a *T*_*C*_ about 750 K and possesses an average magnetic moment of 1.45 *μ*_B_ per magnetic ion in the system (i.e., for both Fe and Mn). This could be achieved due to the strain induced by careful fine tuning of the growth temperature. A thermally induced structural change should be most effective if the structural transition temperature (*T*_S_) is directly related to the high ferromagnetic ordering temperature (*T*_C_). This result opens opportunity to study strain of phase transitions of compounds, and research in magnetism has been crucial to progress in the understanding of diverse types of structure transformation. Our experiment data of high temperature ferromagnetism induced via epitaxial strain play a key role for a better understanding of the structural phase transition of CuAu-I type antiferromagnetic materials and the ferromagnetic mechanism of this material categories.

## Methods

### Epitaxial growth

For the growth of Fe_100−*x*_Mn_*x*_ epilayers, we used an MBE system (VG Semicon Model V80) that consisted of growth, analysis, and load-lock chambers and was equipped with RHEED (reflection high energy electron diffraction) capability. The base pressure of the growth chamber was in the 10^−10^ Torr range. After thermal annealing in an As flux at 600 °C for 30 min to remove the surface oxides, we deposited a 300 nm GaAs buffer layer on the GaAs(100) substrate at 500 °C. Standard effusion cells were used for the iron (Fe), manganese (Mn), and gallium (Ga) evaporations, while a cracking effusion cell was used for the arsenic (As) evaporation. The growth temperature and growth rate of the Fe_50_Mn_50_ epilayers were 30~500 °C and 0.05 nm/s, monitored using a quartz crystal microbalance and an ion gauge beam flux monitor respectively. A 10 nm GaAs capping layer was grown on the Fe_100−*x*_Mn_*x*_ films to avoid oxidization in air.

### Characterization

The crystal structure and microstructure of the grown films were investigated using X-ray diffraction (XRD, Model D/max-RC, Rigaku Co., Tokyo, Japan) and high resolution transmission electron microscopy (HRTEM; 9000-NAR, Hitachi, Japan) studies. The chemical bonding states of the film were investigated by x-ray photoelectron spectroscopy (XPS, VG Escalab 250, UK) system with a monochromatic Al *K*α source (1486.6 eV). The Fe and Mn concentrations of the grown thin films were evaluated using the wavelength dispersive X-ray spectroscopy (WDX) with an electron probe microanalyzer (EPMA, JEOL JXA-8900R, Tokyo, Japan) and compositional depth profiles by XPS measurements. The magnetic properties of the Fe_100−*x*_Mn_*x*_ epilayer were characterized macroscopically by superconducting quantum interference device (SQUID, MPMS, Quantum Design) and magneto-optical Kerr effect (MOKE) measurements. The output of the light source, a 1-mW, 633-nm He-Ne laser (JDSU1100), was passed through a photo-elastic modulator (Hinds PEM-80), The magnet (a 400 Varian electromagnet) produced fields of ±5000 Oe. In order to investigate the correlation between magnetization and charge carrier transport, we performed magnetoresistance (MR) and Hall effect measurements by using a physical property measurement system (PPMS, Quantum Design).

## Supplementary information

Induced high-temperature ferromagnetism by structural phase transitions in strained antiferromagnetic γ-Fe50Mn50 epitaxial films
