# Supplementary material for: Induced high-temperature ferromagnetism by structural phase transitions in strained antiferromagnetic γ-Fe50Mn50 epitaxial films
Source: Sci Rep. 2019 Mar 6;9:3669. doi: 10.1038/s41598-019-39949-x (PMC6403386; doi:10.1038/s41598-019-39949-x)
Supplement: Supplementary file 1 — Induced high-temperature ferromagnetism by structural phase transitions in strained antiferromagnetic γ-Fe50Mn50 epitaxial films [file 41598_2019_39949_MOESM1_ESM.pdf]

Supplementary Information:

Induced high-temperature ferromagnetism by structural phase transitions in strained antiferromagnetic γ-$Fe_{50}Mn_{50}$ epitaxial films

Younghun Hwang,[1] Sungyoul Choi,[2] Jeongyong Choi,[2] and Sunglae Cho,[2,*]

[1]Electricity & Electronics and Semiconductor Applications, Ulsan College, Ulsan 44610, Republic of Korea

[2]Department of Physics and Energy Harvest-Storage Research Center (EHSRC), University of Ulsan, Ulsan 44610, Republic of Korea

Corresponding Author

*E-mail:slcho@ulsan.ac.kr (S.L.C)

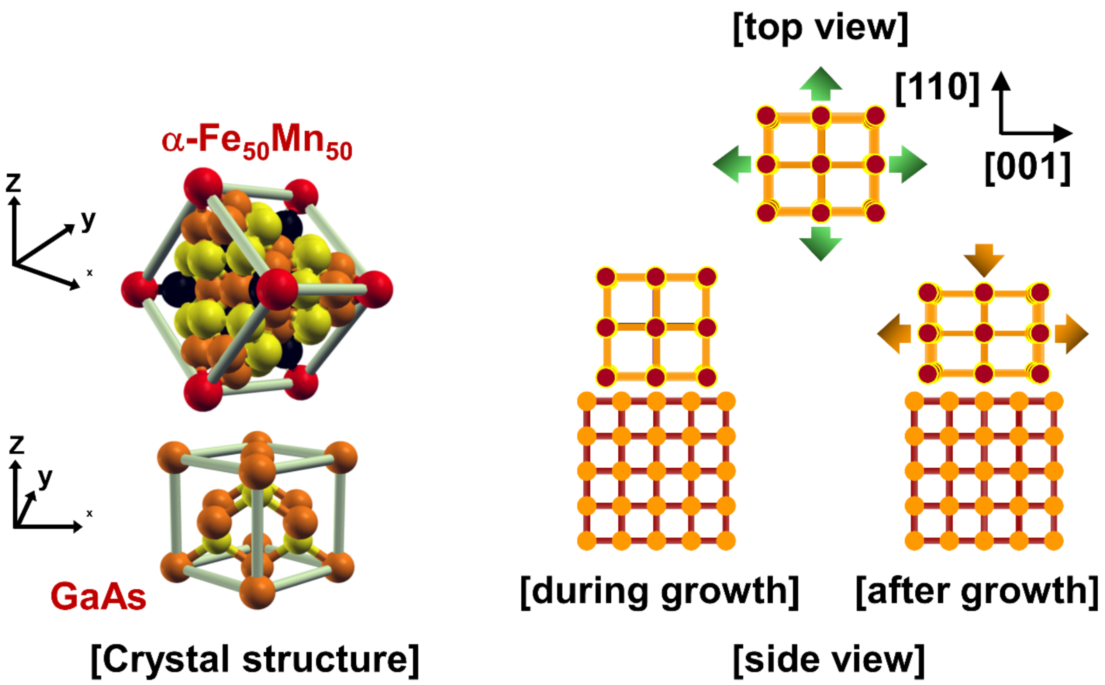

Supplementary Fig. S1 A simple illustration of the tensile strain occurrence in bcc α-phase $Fe_{50}Mn_{50}$ films due to the lattice distortion by difference in thermal expansion coefficients.

Figure S1 illustrates that the $Fe_{50}Mn_{50}$ thin films on GaAs are tensely strained due to the thermal expansion coefficients. The film grows in a volume dependent upon the growth temperature. During growth, the lattice mismatch strain is relaxed in about 5 nm, and when the sample cools down to room temperature after growth, both the film and the substrate are shrunk. Thus, the film is compressed because of the higher film thermal expansion coefficient, which induces a tensile strain.

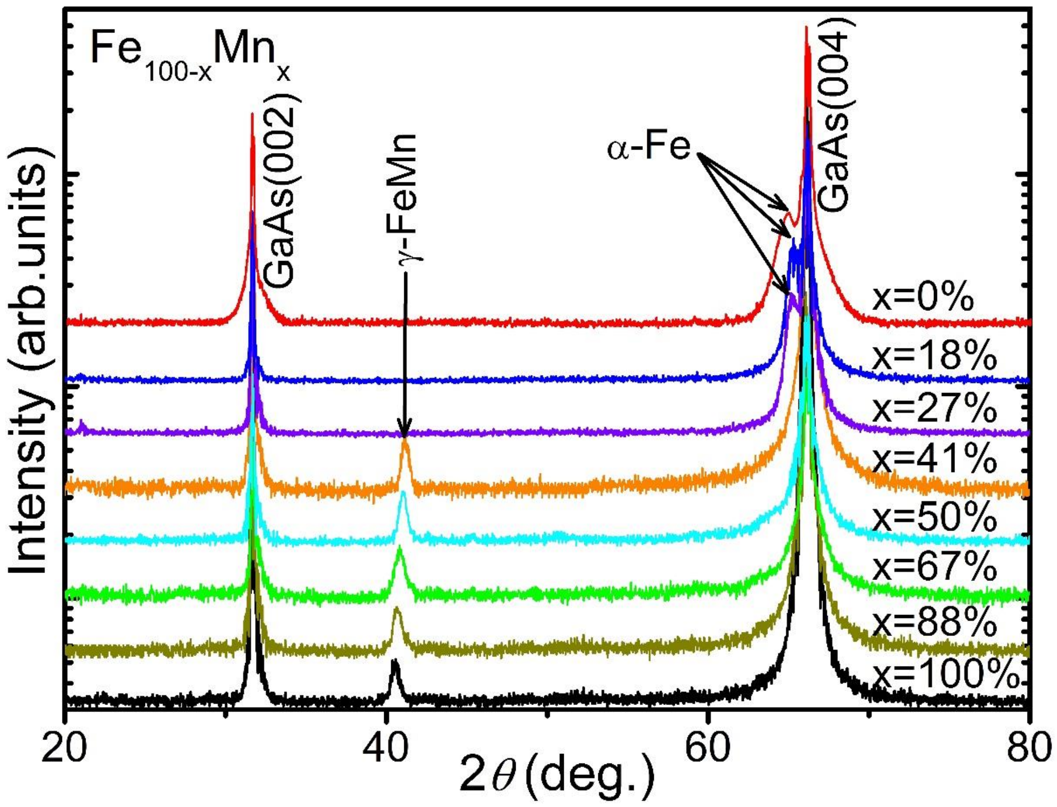

Supplementary Fig. S2 XRD patterns for 100 nm $Fe_{100-x}Mn_x$ films grown at 100°C, the $Fe_{100-x}Mn_x$ films grow in the fcc γ-FeMn phase for the Mn concentration x>40%. Below these limits for x, the α-Fe phase is seen.

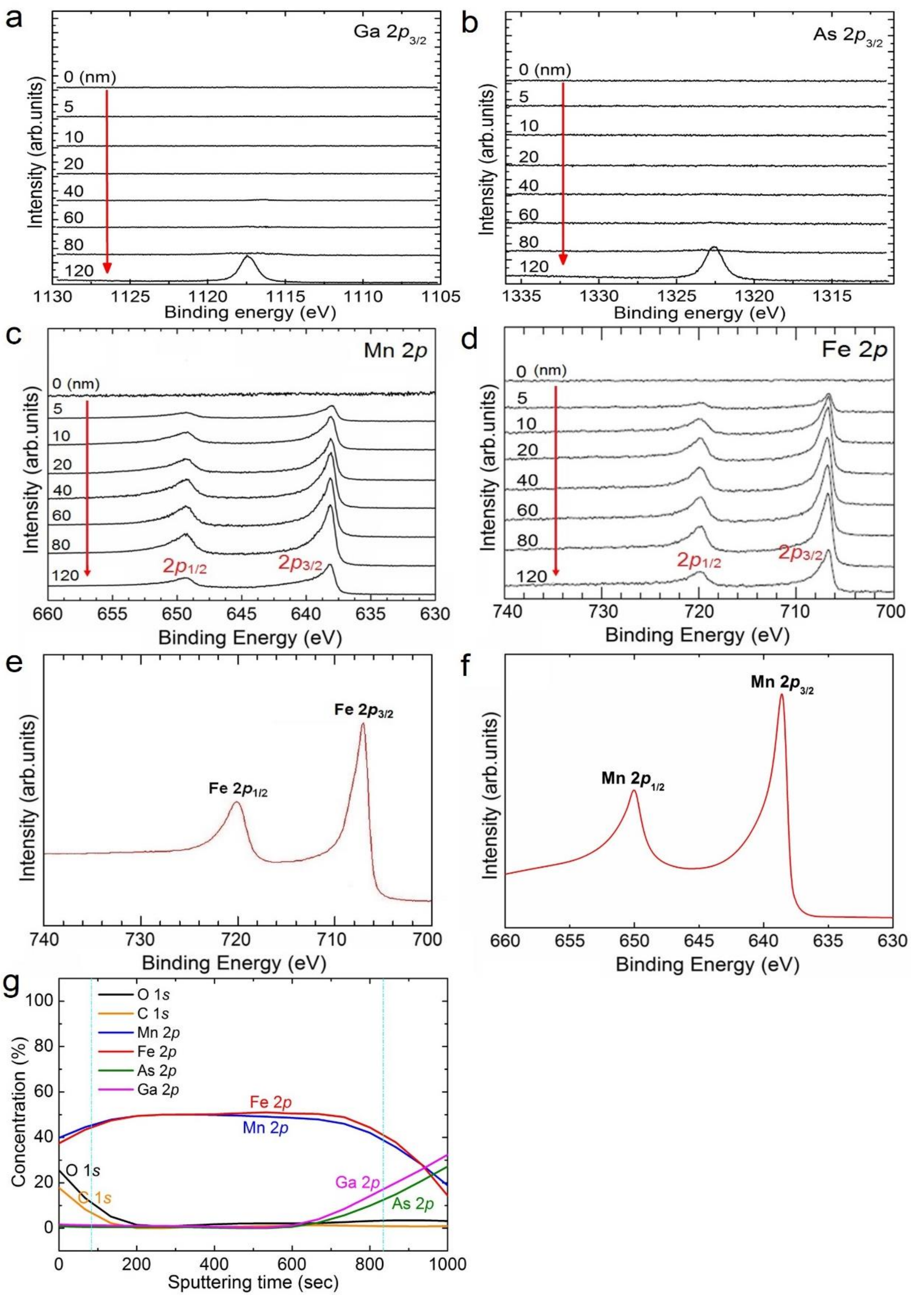

Supplementary Fig. S3 (a) Ga 2$p_{3/2}$ and (b) As 2$p_{3/2}$ spectra at different depths for the α-$Fe_{50}Mn_{50}$ film grown on GaAs at 400°C. (c) Mn 2p and (d) Fe 2p spectra at different depths for the α-$Fe_{50}Mn_{50}$ film grown on GaAs at 400°C. Comparison of the Fe- and Mn-2p XPS spectra of (e) Fe

and (f) Mn metal. (g) XPS compositional depth profiles of α-$Fe_{50}Mn_{50}$ film grown at 400°C with an etch rate of about 1.2 Å /sec. The dashed lines are the real interfacial region between the film and substrate. The thickness of α-$Fe_{50}Mn_{50}$ film is about 100 nm.

Figure S3 shows the Ga 2$p_{3/2}$, As 2$p_{3/2}$, Fe 2p, and Mn 2p spectra at different depths for the α-$Fe_{50}Mn_{50}$ film, after 1 min Ar ion etching to remove any residual surface contamination. The charge shifted spectra were corrected using the adventitious C 1s photoelectron spectra at 285.0 eV. As shown in Figs. S3(a) and S3(b), Ga 2$p_{3/2}$ and As 2$p_{3/2}$ peaks are not seen obviously in the region of a thickness 100 nm less. The results suggest that the Fe and Mn atoms cannot diffuse into a GaAs even at the 400°C. This proves clearly that the ferromagnetism does not originate from FeMnGa, MnAs or GaMnAs phases. Figures S3(c) and S3(d) show the XPS core level spectra of Mn 2p and Fe 2p lines of α-$Fe_{50}Mn_{50}$ film at different depths. The core level peak positions are almost unchanged with increasing depth, and the satellite peaks related to the clusters/oxides are not observed. The metallic Fe and Mn 2p XPS spectrum reveals the usual 2$p_{3/2}$ and 2$p_{1/2}$ doublets arising from spin-orbit splitting (Figs. S3(e) and S3(f)). For metallic manganese the Mn 2$p_{1/2}$ and 2$p_{3/2}$ peaks are located at the center of 650 and 639 eV, respectively. For comparison, peak positions of Mn 2$p_{3/2}$ determined for MnO (Mn^{2+} ions placed with 640.4–641.5 eV), for Mn_2O_3 (Mn^{3+} ions, within 641.1–641.7 eV), and for MnO_2 (Mn^{4+} ions, within 641.1–642.3 eV). In Fe 2p core level, the binding energies for Fe 2$p_{1/2}$ and Fe 2$p_{3/2}$ peaks are about 720.1±0.2 and 706.7 eV at 5 nm depth, respectively. For comparison, the Fe 2$p_{1/2}$ and 2$p_{3/2}$ binding energies have been reported to be near 724.6 and 711.0 eV for Fe^{3+} in Fe_2O_3, 723.17 and 709.53 eV for FeO, 720.1 and 706.9 eV for metallic Fe, as well as 724.07 and 710.56 eV for Fer^{2+} in Fe_3O_4.[12] It can be seen that the Fe 2p lines in the XPS spectra of α-FeMn are quite different that of Fe 2p in iron-oxides, implying that the no oxidation state of the Fe ion on α-$Fe_{50}Mn_{50}$ film. The Mn 2p peaks in the α-$Fe_{50}Mn_{50}$ film spectra are characterized by a Mn 2$p_{3/2}$ and 2$p_{1/2}$ peak with maximum situated at about 638.02 and 649.3±0.2 eV at 5 nm depth, which is similar to the value

of binding energies for metallic Mn (Fig. S3f). The depth XPS results of α-$Fe_{50}Mn_{50}$ film grown at 400°C show that the added Fe into Mn does not form clusters and/or secondary phases, proving a homogeneous α-$Fe_{50}Mn_{50}$ alloy film. This finding is consistent with the results of HRXRD and HRTEM analyses. The results also clearly revealed that the compositional depth distribution of Fe and Mn are homogeneous within the whole thickness of α-FeMn layer.

Figure S3(g) shows the XPS compositional depth profiles of α- $Fe_{50}Mn_{50}$ film grown on GaAs at 400°C. In the Fe-Mn region, the concentration distribution of Fe, Mn, Ga and As atoms is relatively uniform. The atomic percentage of Fe-Mn in this region is >90%, and atomic percentage of Ga and As are both <8%. The compositions of Fe and Mn maintain fairly constant levels expect up to 90 nm for the film surface with GaAs substrate. This results show that the chemical reaction or interdiffusion layers at the interface between Fe-Mn and GaAs did not be observed, proving a homogeneous α-$Fe_{50}Mn_{50}$ alloy film. We found that both concentrations are nearly constant across the film thickness and are approximately 52 at% for Fe and 48 at% for Mn, close to the nominal 50 at% expected for both (within an experimental error of 2 at%), which is consistent with the result of wavelength dispersive X-ray spectroscopy (WDX) with an electron probe microanalyzer (EPMA, JEOL JXA-8900R, Tokyo, Japan).

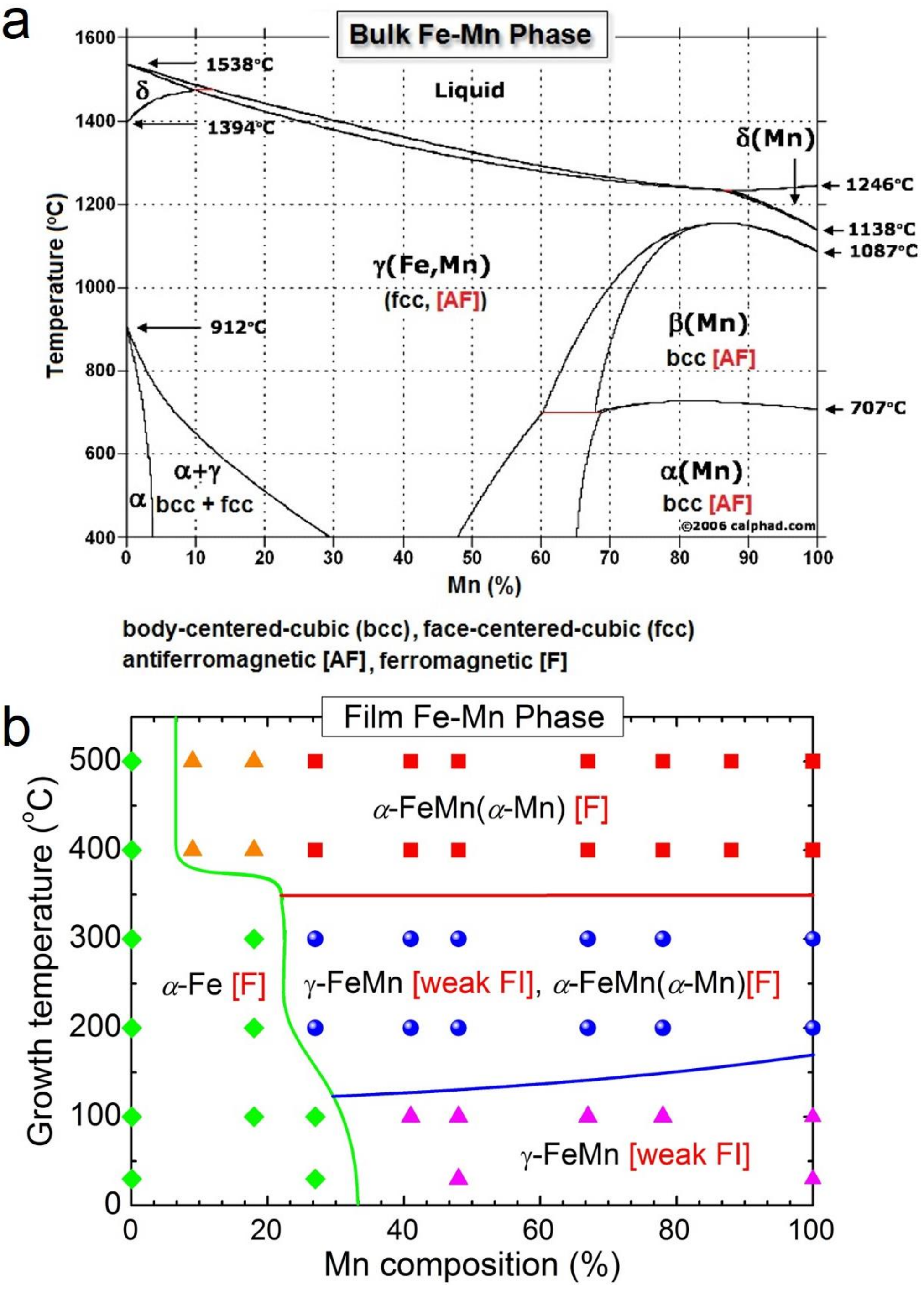

Supplementary Fig. S4 (a) Structural and magnetic phases in the bulk Fe–Mn alloys. (b) A new structural and magnetic phases in epitaxial Fe-Mn alloy films.

References

1. Sun, H. P., Zhang, Z., Wang, W. D., Jiang, H. W. & Lai, W. Y. Microstructure of columnar crystallites in $Ni_{80}Fe_{20}$/Cu magnetic multilayers. *J. Appl. Phys.* **87,** 2835-2839 (2000).

2. Choe, G. & Gupta, S. High exchange anisotropy and high blocking temperature in strongly textured NiFe(111)/FeMn(111) films. *Appl. Phys. Lett.* **70,** 1766-1768 (1997).

3. Hansen, M. &Ardenko, K. *Constitution of Binary Alloys*. (McGraw-Hill, New York, 1958).

4. Paduani, C., da Silva, E. G., Perez-Alcazar, G. A. &McElfresh, M. Mössbauer effect and magnetization studies of α-FeMn alloys. *J. Appl. Phys.* **87,** 7524-7531 (1991).

5. Endoh, Y. & Ishikawa, Y. Antiferromagnetism of γ Iron Manganes Alloys. *J. Phys. Soc. Jpn.* **30,** 1614-1627 (1971).

6. Quang, H. D., Sinh, N. H., Oh, S. K., Huynh, T. N., Hien, T. N., Zidanic, J. & Yu, S. C. Reaction Mechanism of Chemical Elements (Co,Fe,Mn) Existing in Spin Valves Containing Oxides Layers. *Jpn. J. Appl. Phys*. **45,** 88-92 (2006).

7. Yamashita, T. & Hayes, P. Analysis of XPS spectra of Fe^{2+} and Fe^{3+} ions in oxide materials. *Appl. Surf. Sci.* **254,** 2441-2449 (2008).

8. Moulder, J. F., Stickle, W. F., Sobol, P. E. & Bomben, K. *Handbook of X-ray Photoelectron Spectroscopy*. (Perkin-Elmer, Eden Praire, 1995).
